# Upregulation of Notch Signaling and Cell-Differentiation Inhibitory Transcription Factors in Stable Chronic Obstructive Pulmonary Disease Patients

**DOI:** 10.3390/ijms25063287

**Published:** 2024-03-14

**Authors:** Antonino Di Stefano, Isabella Gnemmi, Umberto Rosani, Mauro Maniscalco, Silvestro Ennio D’Anna, Paola Brun, Vitina Carriero, Francesca Bertolini, Bruno Balbi, Fabio Luigi Massimo Ricciardolo

**Affiliations:** 1 Divisione di Pneumologia, Laboratorio di Citoimmunopatologia dell’Apparato Cardio Respiratorio, Istituti Clinici Scientifici Maugeri, IRCCS, Respiratory Rehabilitation Unit of Gattico-Veruno, 28013 Gattico-Veruno, Italy; isabella.gnemmi@icsmaugeri.it (I.G.); centokm@gmail.com (B.B.); 2 Department of Biology, University of Padova, via U. Bassi 58/b, 35121 Padova, Italy; umberto.rosani@unipd.it; 3 Divisione di Pneumologia, Istituti Clinici Scientifici Maugeri, IRCCS, 82037 Telese Terme, Italy; mauro.maniscalco@icsmaugeri.it (M.M.); silvestro.danna@icsmaugeri.it (S.E.D.); 4 Department of Molecular Medicine, Histology Unit, University of Padova, 35121 Padova, Italy; paola.brun@unipd.it; 5 Department of Clinical and Biological Sciences, Severe Asthma and Rare Lung Disease Unit, San Luigi Gonzaga University Hospital, University of Turin, 10043 Orbassano, Italy; vitina.carriero@unito.it (V.C.); francesca.bertolini@unito.it (F.B.); fabioluigimassimo.ricciardolo@unito.it (F.L.M.R.); 6 Azienda Sanitaria Locale (ASL), 28100 Novara, Italy

**Keywords:** Notch signaling, pulmonary rehabilitation, airway inflammation, stem cells, endothelial cells, bronchiolar epithelial cells

## Abstract

Notch signaling is involved in the prevention of cell differentiation and cell fate in various organs, including the lungs. We aimed to determine the transcriptomic and protein expression of Notch receptors, their ligands, and related transcription factors in stable COPD. The expression and localization of Notch receptors, their ligands, and related transcription factors were measured in bronchial biopsies of individuals with stable mild/moderate (MCOPD) (n = 18) or severe/very severe (SCOPD) (n = 16) COPD, control smokers (CSs) (n = 13), and control nonsmokers (CNSs) (n = 11), and in the lung parenchyma of those with MCOPD (n = 13), CSs (n = 10), and CNSs (n = 10) using immunohistochemistry, ELISA tests, and transcriptome analyses. In the bronchial biopsies, Notch4 and HES7 significantly increased in the lamina propria of those with SCOPD compared to those with MCOPD, CSs, and CNSs. In the peripheral lung bronchiolar epithelium, Notch1 significantly increased in those with MCOPD and CSs compared to CNSs. ELISA tests of lung parenchyma homogenates showed significantly increased Notch2 in those with MCOPD compared to CSs and CNSs. Transcriptomic data in lung parenchyma showed increased DLL4 and HES1 mRNA levels in those with MCOPD and CSs compared to CNSs. These data show the increased expression of the Notch pathway in the lungs of those with stable COPD. These alterations may play a role in impairing the regenerative–reparative responses of diseased bronchioles and lung parenchyma.

## 1. Introduction

The Notch signaling pathway influences cell fate decisions, such as survival and apoptosis and cell differentiation and proliferation, and maintains stem-cell quiescence [1,2,3,4]. In mammalians, there are four Notch receptors (Notch1–4) and five ligands (Jagged (Jag) 1 and 2; Delta-like (Dll) 1, 3 and 4) [2,3,4,5]. Ligand–receptor binding is followed by a cascade of cytoplasmic events, followed by the nuclear translocation of a stimulatory complex capable of inducing the transcription of a group of inhibitory transcription factors including HES1,2, 5 and HEY1,2 and L, capable, in turn, of suppressing differentiation factors such as ASCL1, UBE2A, and MYOD [2,3,4,5]. Therefore, when Notch signaling is active, it may suppress the transcription of differentiation factors [2,3,4,5].

A progressive reduction in the number of peripheral airways has been reported in patients with COPD [6,7,8,9], where this airway reduction becomes evident from the ninth airway generation to the peripheral lung [6,7,8,9]. These molecular alterations are also present, to a lesser extent, in smokers with near-normal lung function [7,8,9] who develop COPD. Variable degrees of emphysema and lung alveolar septa reduction are also reported in susceptible smokers and patients with COPD [6,7,8,9].

Concerning Notch signaling levels, conflicting results are reported in patients with COPD. In fact, data showing decreased [10,11] or increased [12] lung protein Notch signaling expression, in comparison with controls, have been reported. Apparently, different functional results are also reported in animal models, where Notch1’s increase [13] or its decrease [14] may induce increased goblet cell metaplasia and the decreased presence of ciliated cells, and it may interfere with the differentiation of alveolar cell types [13,14].

In vitro studies showed that in the mouse retina inhibition of Notch signaling, the inactivation of the DLL4 ligand or the deletion of the Notch1 receptor increases the number of tip cells and angiogenesis [15], and the activation of Notch by the Jag1 ligand reduces tip cells and vessel branch formation [15]. In developing segmental arteries in zebrafish embryos, Notch activation reduces angiogenesis, and DLL4 ligand decrement leads to an increased number of endothelial cells [16]. In zebrafish, Notch signaling is responsible for limiting blood vessel growth during venous plexus formation after an initial stimulating role developed during artery formation [17].

The Notch system has also been reported to play a regulative role in Th1, Th2, and Th17 immune response and after mycobacterial challenge [18,19]. Also, viral infections in mice indicate that DLL1 ligand responses and IFNγ increases play roles in combatting infection [18,20,21].

We hypothesized that altered Notch pathway expression within the bronchial mucosa and lung parenchyma could be associated with the worsening of COPD. We therefore examined the expression of Notch receptors, their ligands, and effector molecules in bronchial biopsies and peripheral lungs of COPD patients and healthy controls using immunohistochemistry, ELISA tests, and transcriptomic analyses. We also used an in vitro epithelial cell model to examine the impact of bacterial or oxidative challenges on the regulation of Notch signaling molecules in order to better define the potential role of this potentially regenerative–reparative molecular pathway. 

## 2. Results

### 2.1. Clinical Characteristics of Subjects Providing Bronchial Biopsies and Lung Parenchyma

We obtained and studied bronchial biopsies from 58 subjects: 34 with mild/moderate (MCOPD) and severe–very severe (SCOPD) stable COPD; 13 were current or ex-smokers with normal lung function and 11 were lifelong nonsmokers with normal lung function (Table 1). Peripheral lung samples were obtained from the lung resections of 33 subjects, whose characteristics are shown in Table 2. Thirteen of these had COPD, while the other twenty had normal lung function. Half of the control subjects were smokers.

### 2.2. Immunohistochemistry for Notch Signaling Proteins in the Bronchial Epithelium of Bronchial Biopsies

**Figure 1:**
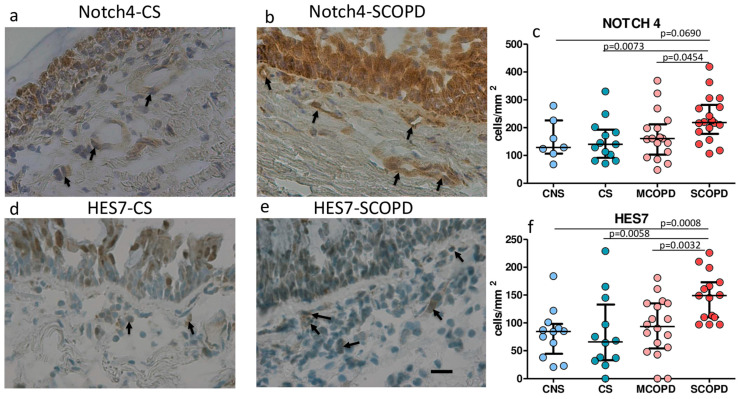
Photomicrographs showing the bronchial mucosa from (**a**,**d**) control nonsmokers, (**b**,**e**) severe/very severe stable COPD patients immunostained for identification of Notch4+ cells (**a**,**b**) and HES7+ cells (**d**,**e**) in the epithelium (E) and bronchial lamina propria. Results are representative of those from 11 nonsmokers and 16 with severe/very severe COPD patients. The arrows indicate immunostained endothelial cells, inflammatory cells, and fibroblasts in the lamina propria of a patient with severe/very severe COPD and a control smoker with normal lung function. Bar = 20 micron. Panels (**c**,**f**) show the quantitative analyses performed in lamina propria for immune expression of Notch4 (**c**) and HES7 (**f**). Data are expressed as median (IQR).

No statistically significant differences in the expression of Notch signaling molecules were seen in the bronchial epithelium of COPD patients compared to nonsmoking and smoking controls. The most expressed molecules, exceeding one-third of immunostained bronchial epithelial cells (scored value ≥ 1), were Jagged2, Notch4, HES1,3,5,7, and HEY2 (Table 3, Figure 1).

### 2.3. Immunohistochemistry for Notch Signaling Proteins in the Bronchial Lamina Propria of Bronchial Biopsies

No significant differences in Jagged1, Jagged2, Delta1, Notch1–3, HES1, HES3, HES6, HEY2, and HEYL expression were observed in the lamina propria across groups (Table 3). Notch4 and HES7 immunostained cells were significantly increased in the bronchial lamina propria of SCOPD patients compared to MCOPD, CNS, and CS (Table 3, Figure 1). The immunopositivity for Notch4 and HES7 was mainly observed in endothelial cells and, to a minor extent, in fibroblasts and inflammatory cells (Figure 1). Relatively high levels of immunopositivity were also observed for HEY2, HES5, HES1, and Jagged2 in lamina propria, even though the difference between groups was not statistically significant (Table 3).

### 2.4. Immunohistochemistry for Notch Signaling Proteins in the Peripheral Airways and Lung Parenchyma

The shortest internal diameters of the bronchioles studied, as measured in the H&E-stained sections from each patient, were (mean ± SD) 268 ± 56, 289 ± 57, and 263 ± 81 μm, respectively, in CNS, CS, and patients with COPD. On average, the numbers of peripheral bronchioles studied in the three groups of subjects were 10.33 ± 5, 7.83 ± 3.8, and 5.8 ± 2.5, respectively, in CNS, CS, and in patients with COPD.

We examined bronchiolar epithelial cells, bronchiolar lamina propria, alveolar macrophages, alveolar septa, and lung vessels. In bronchiolar epithelium, increased levels of Notch1 immunopositivity was observed in MCOPD and in CS when compared to CNS (Table 4). More than one-third (scored value ≥ 1) of bronchiolar epithelial cells were positively stained for Notch4, HES1, and HES5 in MCOPD and CS. In bronchiolar lamina propria, no significant differences were observed between the groups. The most expressed molecules (scored value ≥ 1) were Notch4 and HES5 in MCOPD. In the alveolar macrophages, Delta4 and Notch2 were significantly increased in CS compared to CNS (Table 4), while this difference was not statistically significant when compared with MCOPD vs. CNS. The most expressed molecules (scored value ≥ 1) were Notch4, HES5, and HES7 in all three groups studied. In alveolar septa, no significant differences were observed between the groups for all the Notch signaling molecules studied. The most expressed molecules (scored value ≥ 1) were Notch4 and HES5 in this lung compartment (Table 4). In lung vessels, no significant differences were observed between the groups. The most expressed molecules (scored value ≥ 1) were Noth4 and HES5. Endothelial cells were the principal positive cells expressing all molecules.

### 2.5. ELISA Tests for Notch Signaling Proteins in Homogenized Peripheral Lung Tissue

**Figure 2:**
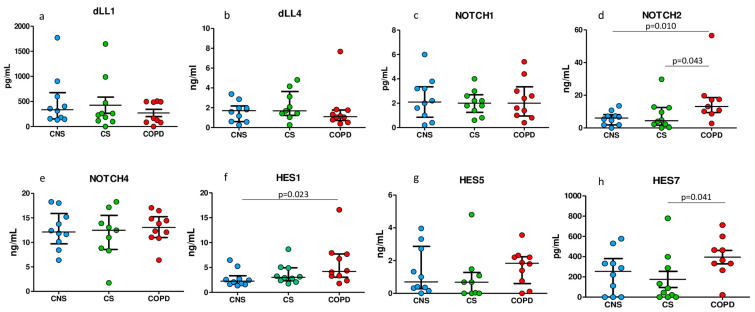
ELISA tests were performed in frozen lung tissue homogenates of patients with MCOPD (n = 13), CS (n = 10), and CNS (n = 10) for quantification of Notch receptors, their ligands, and their effector transcription factors. The data show increased levels of Notch2 in MCOPD compared to CS and CNS (panel (**d**)), increased level of HES1 in MCOPD vs. CNS (panel (**f**)), and increased HES7 in MCOPD vs. CS (panel (**h**)). DLL1, DLL4, Notch1 (panels (**a**–**c**)), Notch4, and Hes5 (panels (**e**,**g**)) were not significantly changed. The Mann–Whitney U-test was applied for comparison between groups. CNS, control nonsmokers; CS, control smokers with normal lung function. Data are expressed as median (IQR).

As shown in Figure 2, we found significant differences in the concentrations of Notch2, HES1, and HES7 between groups. Notch2 was significantly increased in MCOPD compared to CS and CNS subjects (Figure 2d). HES1 was significantly increased in MCOPD compared to CNS (Figure 2f). HES7 was significantly increased in MCOPD compared to CS (Figure 2h). No significant differences were observed for the other molecules studied. ELISA tests also confirmed that the most expressed molecules in the lung parenchyma were Notch2, Notch4, HES1, and HES5 proteins, as was similarly observed by immunohistochemistry.

### 2.6. Gene Expression Level of Notch Signaling Molecules in Bronchial Rings and Lung Parenchyma

**Figure 3:**
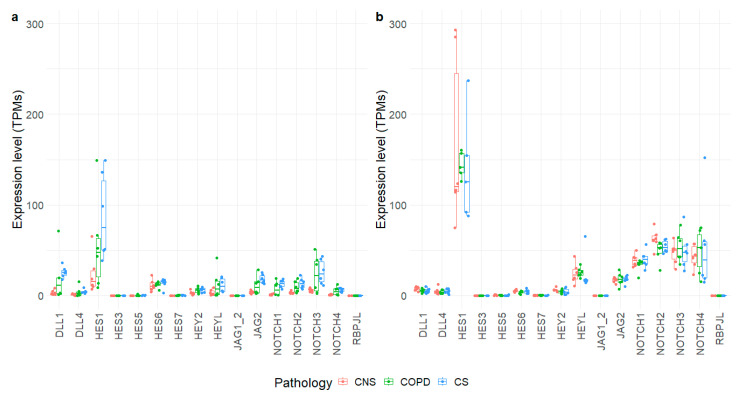
The expression levels of 16 selected genes obtained from bronchial rings (**a**) and lung parenchyma (**b**) of control nonsmokers (CNS), control smokers (CS), patients with chronic obstructive pulmonary disease (COPD). The box plot shows the median and the distribution of expression values per gene reported as transcripts per million (TPMs).

We examined RNA-seq expression data for 16 selected Notch signaling molecules in the lung parenchyma from frozen blocks used for immunohistochemical analysis and from frozen bronchial rings (Table S2 and Figure 3). In bronchial rings, no significant modulations of these genes was observed, although HES1 showed higher expression levels in COPD and, particularly, in CS samples (Figure 3a). Pertaining to the four considered NOTCH transcripts (NOTCH1–4), they appeared slightly modulated with expression levels below 50 TPMs. Conversely, in lung parenchyma, the NOTCH transcripts appeared more modulated, with NOTCH1 and NOTCH4 showing statistically significant increases in COPD and CS samples compared to CNS ones (Figure 3b and Table S2). In this second dataset, the most expressed transcripts were shown to be HES1, confirming the high expression, also observed in bronchial rings. All the other tested molecules did not show significant modifications of their mRNA expression levels.

### 2.7. ELISA Tests for Notch Signaling Molecules in the LPS- and H_2_O_2_-Treated and Nontreated Lysed 16HBE Cells

**Figure 4:**
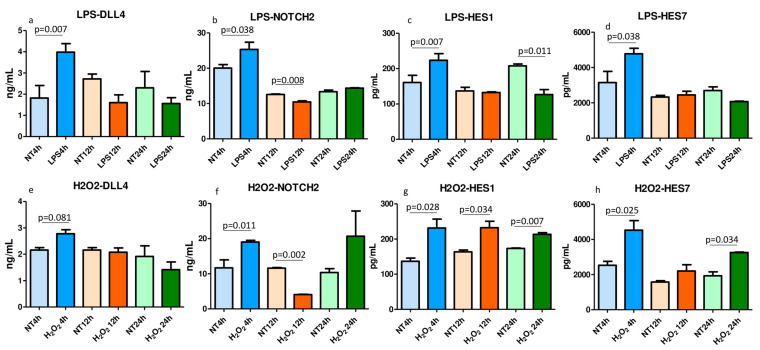
In vitro quantification by ELISA tests of DLL4, Notch2, HES1, and HES7 after LPS (10 µg/mL) (**a**–**d**) and H_2_O_2_ (100 µM) (**e**–**h**) stimulation of 16HBE cells. Cell lysates were used for quantification of Notch signaling molecules. The challenges with LPS significantly increased DLL4 (**a**), Notch2 (**b**), HES1 (**c**), and HES7 (**d**) at 4 h after stimulation. H_2_O_2_ significantly upregulated Notch2 (**f**), HES1 (**g**), and HES7 (**h**) at 4 h after stimulation. HES1 was also upregulated at 12 and 24 h (**g**). HES7 was also significantly increased at 24 h (**h**) after the H_2_O_2_ challenge. The experiments were performed in quadruplicate. The *t*-tests were applied for comparison between groups. Data are expressed as mean ± SEM.

Human bronchial epithelial cells (16HBE) were treated with LPS, 10 µg/mL, and H_2_O_2_, 100 µM, for up to 24 h (Figure 4). DLL1, DLL4, Notch1, Notch2, Notch4, HES1, HES5, and HES7 concentrations were quantified in nontreated (NT) and LPS- and H_2_O_2_-treated cells at 4 h, 12 h, and 24 h after challenge. The LPS challenge significantly upregulated DLL4 (Figure 4a), Notch2 (Figure 4b), HES1 (Figure 4c), and HES7 (Figure 4d) at 4 h after challenge. H_2_O_2_ stimulation significantly upregulated Notch2, HES1, and HES7 at 4 h (Figure 4f–h), HES1, also at 12 h and 24 h (Figure 4g), and HES7, also at 24 h (Figure 4h) after challenge. DLL4 tended to increase at 4 h after challenge (Figure 4e, *p* = 0.081). All the other molecules were not significantly changed after LPS or H_2_O_2_ challenges. These data show that bacterial challenges (LPS) and oxidative stress (H_2_O_2_) are both able to stimulate the upregulation of the Notch signal molecules at different levels of the Notch pathway.

## 3. Discussion

We report here the localization and a different expression of Notch signaling molecules, their ligands, and related transcription factors in bronchial biopsies and in peripheral lungs of patients with stable COPD compared to control subjects. At all the levels of the lung, we observed upregulation of the Notch signaling molecules. In bronchial biopsies, Notch4 and HES7 significantly increased in the lamina propria of SCOPD compared to MCOPD, CS, and CNS patients. In peripheral lungs, Notch1, in bronchiolar epithelium, in MCOPD and CS, compared to CNS, increased significantly. In alveolar macrophages, Notch2 and DLL4 increased significantly in CS compared to CNS. ELISA tests of lung parenchyma homogenates showed significantly increased levels of Notch2 in MCOPD compared to CS and CNS patients. Transcriptomic data from bronchial rings supported the modulation of Notch molecules, particularly in bronchial rings. In vitro stimulation of 16HBE cells with LPS induced a significant increase in DLL4, Notch2, HES1, and HES7 at 4 h after challenge. H_2_O_2_ stimulation significantly upregulated HES1 and HES7 at 4 h and 24 h and Notch2 at 4 h after challenge.

In bronchial epithelium of the bronchial biopsies, we observed no significant variations of the Notch signaling molecules; this was at variance with the peripheral airways of the bronchiolar compartment, where Notch1 showed an increase in MCOPD and in CS compared to CNS patients. This result was also supported by transcriptomic data. In bronchial lamina propria, Notch4 and HES7 were increased, particularly in SCOPD, compared to all the other groups. This immunopositivity found in lamina propria was mainly due to immunostained endothelial cells, fibroblasts, and inflammatory cells, all of which are cellular types identifiable by morphology and location in the lamina propria. These findings show that bronchial and bronchiolar epithelium differ, in part, in expressing Notch signaling molecules. HES1, as confirmed in a previous work [10], were abundantly expressed in bronchial and also in bronchiolar epithelium, as here reported, even though they were not significantly upregulated in COPD and showed very significant expression levels, whereas, in the peripheral airways and in the lamina propria of bronchial biopsies, Notch4 and HES7 were upregulated. This finding can be attributed to inflammatory cells and macrophage immunostaining populating the bronchial lamina propria, which are increased [22,23] particularly in SCOPD patients. These data from bronchial biopsies may suggest a regulative role of Notch signaling molecules in driving the inflammatory response through a Tc1 type in patients with COPD, as previously suggested [21,24,25].

Interestingly, in the present study, alveolar macrophages showed increased levels of the DLL4 ligand and of Notch2 receptor, particularly in CS compared to CNS patients, showing that smoking habits may directly influence the level of immunoexpression of the Notch signaling molecules.

ELISA tests applied in lung tissue homogenates, capable of quantifying the total amount of proteins coming from different lung compartments, confirmed the upregulation of the Notch system in the peripheral lungs of MCOPD patients, showing upregulation of Notch2 and HES1 in MCOPD when compared to control nonsmokers.

This is at variance with Tilley’s study [10], where we noted an increase in DLL1 mRNA in bronchial rings and increased levels of DLL4 and HES1 mRNA in peripheral lungs of MCOPD patients compared to control nonsmokers. This discrepancy may be due to the different methods used for mRNA quantification, or to different stage and clinical characteristics of patients studied. Moreover, we focused our attention on protein-level analyses, performed in different lung compartments, whereas in the Tilley’s study, only a few molecules were studied by immunohistochemistry in bronchial biopsies. However, in agreement with our data, HES1 and HES5 proteins were abundantly and similarly expressed in bronchial biopsies in both ours and Tilley’s studies, among the three groups (COPD, CS, CNS) [10].

Looking at the main functions of Notch signaling molecules in the lungs, in a mouse retina, the activation of Notch reduces tip cells and vessel branch formations [15,16]. Furthermore, in zebrafish, Notch signaling limits blood vessel growth during venous plexus formation [17]. Goblet cell metaplasia in lung airways of COPD patients has been associated with Notch1 and HEY2 increase in bronchial epithelial cells and in submucosal glands [12]. In Boucherat’s study, in part, in agreement with our present work, increased levels of Notch signaling molecules were reported in COPD patients, even though its formal quantification was not performed in that study [12]. These data, taken together, may suggest a role for Notch signaling molecules in the pathogenesis of COPD patients, particularly in the reduction in the regenerative–reparative response of peripheral airways and lung alveolar septa (6–9) and in favoring goblet cell metaplasia [12].

In our present study, in vitro stimulation of 16HBE cells with LPS induced a significant increase in ligands, receptors, and the related transcription factors, DLL4, Notch2, HES1, and HES7, and H_2_O_2_ stimulation significantly upregulated HES1, HES7, and Notch2. In previous “in vitro” studies, conducted in 16HBE cells treated with H_2_O_2_ and LPS, used at the same concentrations as in the present study, the expression of IL-8 and p65 NF-kB subunit mRNA after H_2_O_2_ and of IL-8 mRNA after LPS were upregulated after challenges [26]. H_2_O_2_ also significantly increased IL-27B mRNA and protein expression [22] and TLR4 mRNA [27] in other studies. These data show that in bronchial epithelial cells, bacterial and oxidative stress challenges upregulate not only the inflammatory molecules related to COPD patients, but also molecules belonging to the Notch pathway.

The Notch system has also been reported as playing a regulative role in Th1, Th2, and Th17 immune responses [10]. An accessory effect of the Notch system has been reported in Th1 cell differentiation [28]. During mycobacterial challenges, overexpression of DLL4 augmented IL-17 production by CD4+ T cells [19]. The DLL4 increase may induce a Th2 to Th1 shift response in cultured T cells [29]. Viral infections in mice indicate a role for DLL1 ligand response and IFNγ increase in countering these viral infections [18,20,21]. We hypothesize that the Notch system, which is upregulated in the lungs of COPD patients, as here reported, may have a role in regulating lung inflammation in these patients.

This work has some strengths and limitations. Importantly, the present study supports the upregulation of the Notch system in the large and, particularly, small airways and lung parenchyma of COPD patients and, to a lesser extent, in control smokers compared to control nonsmokers. This result, in fact, has been obtained using different quantitative or semi-quantitative approaches at protein and mRNA levels, shedding light on the presence of conflicting results previously reported in the literature. On the contrary, mechanistic actions of the Notch system, in relation to its specific regenerative–reparative or inflammatory-regulative functions, have not been studied directly in this work. As a consequence, our observations on potential specific functions of these upregulated molecules are reported as hypotheses. However, our “in vitro” findings show a possible link between specific bacterial or oxidative challenges, stimulating the bronchial epithelial cells and the upregulation of the Notch pathway.

## 4. Materials and Methods

### 4.1. Subjects

All COPD and healthy control subjects were recruited from the Respiratory Medicine Unit of the “Istituti Clinici Scientifici Maugeri” (Veruno, Italy) and the San Luigi Gonzaga University Hospital (Orbassano-Turin, Italy). Archival material was used in the present study [22]. The characteristics of these subjects that were used for analysis of bronchial biopsies and lung parenchyma are reported in Table 1 and Table 2, respectively. The study conforms to the Declaration of Helsinki and the study was approved by the Institutional Review Boards of Istituti Clinici Scientifici Maugeri (protocol p112) and by Ethical Committee of the San Luigi Gonzaga University Hospital (protocol n. 9544/2019). More details on the characteristics of the subjects are reported in Supplementary Materials.

### 4.2. Lung Function Tests and Volumes

Pulmonary function tests were performed on all subjects, according to current guidelines. The severity of the airflow obstruction in COPD patients was staged using GOLD criteria [https://goldcopd.org/wp-content/uploads/2021/12/GOLD-REPORT-2022-v1.1-22Nov2021_WMV.pdf] (accessed on 11 March 2024) on the basis of pulmonary function tests. More details are reported in Supplementary Materials.

### 4.3. Fiberoptic Bronchoscopy, Collection and Processing of Bronchial Biopsies

Bronchial biopsies for immunohistochemistry were obtained from the patients and processed for light microscopy [22]. At least two samples were embedded in Tissue Tek II OCT, frozen within 15 min in isopentane precooled in liquid nitrogen, and stored at −80 °C. The best frozen sample was then oriented and 6 μm thick cryostat sections were cut for immunohistochemical light microscopy analysis and processed as described below.

### 4.4. Collection and Processing of the Peripheral Lung Tissue

Collection and lung tissue processing was performed as previously described [22,23]. Two to four randomly selected tissue blocks and one to two bronchial rings were taken from the lung obtained at surgery, avoiding areas grossly invaded by tumor. Frozen tissue specimens were used in this study. Specimens were then cut for immunohistochemical analysis and were placed on charged slides, as previously reported [22,23].

### 4.5. Immunohistochemistry on OCT-Embedded Bronchial Biopsies

Sections from each sample were stained with antibodies specific for Notch signaling molecules, their ligands, and their effector transcription factors (Table S1). For the negative control, nonspecific immunoglobulins were used at the same protein concentration as the primary antibody. Details of the methods used are reported in Supplementary Materials.

### 4.6. Immunohistochemistry in Human Peripheral Lung Tissue

Immunostaining of frozen peripheral lung tissue was performed as previously described [22,23]. In the present study, frozen sections were used for immunohistochemical analysis.

The sections were immunostained with primary antibodies used for bronchial biopsies (Table S1). Control slides were included in each staining run using human tonsils or nasal polyps as a positive control for all the immunostaining performed. More details are reported in Supplementary Materials.

### 4.7. Scoring System for Immunohistochemistry in the Bronchial Biopsies

Light-microscopic analysis was performed at a magnification of 630×. The immunostaining for all the antigens studied was scored in the intact bronchial epithelium. Immunostained cells in the bronchial lamina propria were quantified 100 μm beneath the epithelial basement membrane.

### 4.8. Scoring System for Immunohistochemistry in the Peripheral Lung Tissue

All disposable bronchioles, alveolar macrophages, alveolar septa, and vessels observed in each lung section specimen were analyzed for each immunostained section. The immunopositivity was scored as previously described [22,23]. More details for scoring system in bronchial biopsies and lung tissue are reported in Supplementary Materials.

### 4.9. ELISA Tests in the Peripheral Lung Tissue Homogenates

DLL1, DLL4, Notch1, Notch2, Notch4, HES1, HES5, and HES7 protein quantification was performed in the lung tissue homogenates obtained from frozen tissue specimens, which were also used for immunohistochemical analysis. More details are reported in Supplementary Materials.

### 4.10. RNA Extraction and Sequencing from Bronchial Rings and Lung Specimens

The frozen lung parenchymal tissue used for immunohistochemical analysis and bronchial rings from the same patients was also used for RNA extraction, sequencing, and gene expression data analyses.

### 4.11. Data Analysis of RNA-Seq Data

The raw Illumina reads were trimmed for quality using fastp [30], setting a minimal Phred quality of 25 and removing the sequencing adaptors. Raw Illumina datasets have been submitted to the NCBI Short Read Archive (SRA) under the project ID PRJNA1041288. Expression values were counted as transcripts per million (TPMs). Limited to the gene of interest, the expression levels were extracted from the overall dataset and further discussed. More details on RNA extraction, sequencing, and data analysis are reported in Supplementary Materials.

### 4.12. Cell Culture and Treatments

We used the 16HBE cell line (normal human bronchial epithelial cells (NHBE) [31] for “in vitro” experiments. The 16HBE cells were cultured for 0–24 h. Nontreated 16HBE cells were used as controls. All experiments were performed in quadruplicate for each type of treatment (LPS, H_2_O_2_) and each time exposure (4–12–24 h).

### 4.13. ELISA Tests in the Cell Lysates of LPS- and H_2_O_2_-Treated and Untreated 16HBE Cells

DLL1, DLL4, Notch1, Notch2, Notch4, HES1, HES5, and HES7 proteins were also analyzed in treated and untreated 16HBE cells using the same ELISA kits as reported for peripheral lung tissue homogenates. More details for cell cultures and ELISA tests in the cell lysates are reported in Supplementary Materials.

### 4.14. Statistical Analysis Applied to Functional and Morphological Data

The differences between groups were analyzed using analysis of variance (ANOVA) for functional data or Kruskal–Wallis test for morphologic data, followed by a Mann–Whitney U-test. Probability values of *p* < 0.05 were considered significant. Data analysis was performed using the Stat View SE Graphics program. More details are reported in Supplementary Materials.

## 5. Conclusions

Our present data show an increased expression of the Notch pathway in the lung, proximal and peripheral airways, and lung parenchyma of stable COPD patients. These alterations may play a role in impairing the regenerative–reparative responses of the diseased bronchioles/alveoli and in directing the inflammatory response in COPD patients.

## Figures and Tables

**Table 1:** Clinical characteristics of subjects for immunohistochemistry studies of bronchial biopsies.

Groups	n	Age (years)	M/F	Pack Years	Ex/Current Smokers	FEV_1_ (% pred) pre-β_2_	FEV_1_ (% pred) post-β_2_	FEV_1_/FVC (%)
Control nonsmokers	11	64 ± 11	10/1	0	0	111 ± 16	ND	84 ± 9
Control smokers with normal lung function	13	60 ± 8	9/4	40 ± 9	2/11	101 ± 14	ND	81 ± 5
COPD grades I and II (mild/moderate)	18	68 ± 9	15/3	46 ± 6.3	6/12	66 ± 13 ^#^	70 ± 12	58 ± 9 ^#^
COPD grades III and IV (severe/very severe)	16	66 ± 12	11/5	61 ± 11	13/3	36 ± 7 ^#&^	41 ± 6	44 ± 9 ^#&^

Data are expressed as mean ± SE. Patients with chronic obstructive pulmonary disease (COPD) were classified according to GOLD 2011 (goldcopd.org) grades of severity only using the severity of airflow obstruction. For COPD patients, FEV_1_/FVC (%) are post-bronchodilator values. Abbreviations: M, male; F, female, FEV_1_: forced expiratory volume in one second; FVC, forced vital capacity; ND, not determined; statistical analysis: ANOVA test; ^#^, *p* < 0.0001, significantly different from control smokers with normal lung function and controls who have never smoked; ^&^, *p* < 0.0001, significantly different from mild/moderate COPD.

**Table 2:** Clinical characteristics of subjects for immunohistochemistry studies on the peripheral lung tissue.

Groups	N°	Age (year)	M/F	Ex/Current Smokers	Pack Years	FEV1 (% pred) pre-β_2_	FEV_1_ (% pred) post-β_2_	FEV_1_/FVC (%)
Control nonsmokers	10	71 ± 3	5/5	---	---	116 ± 5.1	ND	80 ± 1.6
Control smokers	10	68 ± 1.7	7/3	7/3	38 ± 4.3	96 ± 2.6	ND	74 ± 1.4
Patients with COPD	13	70 ± 1.3	11/2	11/2	61 ± 8.8	74 ± 3.3 ^#^	81 ± 3.7	62 ± 1.3 ^#^

Data are expressed as mean ± SE. Patients with chronic obstructive pulmonary disease (COPD) were classified according to GOLD 2011 (goldcopd.org) grades of severity using only the severity of airflow obstruction. The values for COPD patients FEV_1_/FVC (%) are post-bronchodilator values. Abbreviations: M, male; F, female; FEV_1_: forced expiratory volume in one second; FVC: forced vital capacity; ND, not determined; statistical analysis: ANOVA test; ^#^, *p* < 0.0001, differ significantly from control smokers with normal lung function and controls who have never smoked.

**Table 3:** Immunohistochemical quantification of Notch signaling proteins in bronchial biopsies.

	Healthy Nonsmokers	Healthy Smokers	Mild/Moderate COPD	Severe COPD	Kruskal–Wallis *p*-Value
**Bronchial Epithelium (score 0–3)**	Median (Range)	Median (Range)	Median (Range)	Median (Range)	
JAGGED 1	0 (0–0.25)	0 (0–0)	0 (0–0)	0 (0–0)	0.947
JAGGED 2	1.25 (0.5–2.75)	2 (0.5–3)	1.13 (0.5–3)	1.75 (0.5–2.75)	0.244
DELTA 1	0 (0–0)	0 (0–1)	0 (0–0)	0 (0–0)	0.987
DELTA 4	0 (0–0.5)	0 (0–0.25)	0 (0–0.5)	0 (0–0.5)	0.869
NOTCH 1	0 (0–0)	0 (0–0.25)	0 (0–0)	0 (0–0.5)	0.978
NOTCH 2	0 (0–1)	0 (0–1)	0 (0–2)	0 (0–1)	0.884
NOTCH 3	0 (0–0.25)	0 (0–1)	0 (0–0.25)	0 (0–0.5)	0.980
NOTCH 4	1.25 (0–2.5)	2 (1–5)	1.88 (0.5–2.5)	2 (1–3)	0.360
RBP-JK	0.13 (0–0.75)	0 (0–0.25)	0 (0–0.5)	0 (0–0.5)	0.600
HES-1	2 (1–2.5)	1.5 (0.25–3)	1.75 (0.75–2.5)	1.75 (0.5–2.5)	0.695
HES-3	2.37 (1.5–2.75)	2.25 (0.75–3)	2 (1.5–2.5)	2.5 (1.25–3)	0.547
HES-5	3 (2.5–3)	2.5 (1.75–3)	2.5 (2–3)	2.87 (1.75–3)	0.187
HES-6	0.25 (0–0.75)	0.25 (0–0.75)	0 (0–1.25)	0.25 (0–1.5)	0.783
HES-7	0.5 (0.25–1.5)	0.5 (0.25–1.25)	0.5 (0–1.75)	1 (0.25–1.75)	0.179
HEY2	1.5 (1–2)	1 (0.75–2)	1.25 (0.75–2)	1.5 (0.5–2.25)	0.404
HEYL	0.62 (0–1)	0.5 (0–1)	0.5 (0–1)	0.5 (0–0.75)	0.815
**Bronchial Lamina propria (cells/mm^2^)**	Median (Range)	Median (Range)	Median (Range)	Median (Range)	
JAGGED 1	0 (0–8)	2.5 (0–12)	4 (0–25)	0 (0–16)	0.547
JAGGED 2	21 (8–206)	64 (8–166)	37 (5–202)	62 (5–223)	0.470
DELTA 1	0 (0–6)	0 (0–12)	0 (0–7)	3 (0–19)	0.584
DELTA 4	0 (0–11)	0 (0–11)	6 (0–113)&	1.5 (0–38)	0.207
NOTCH 1	0 (0–16)	0 (0–6)	0 (0–9)	0 (0–51)	0.577
NOTCH 2	11 (0–116)	9 (0–113)	9 (0–172)	5.5 (0–123)	0.795
NOTCH 3	1 (0–9)	2.5 (0–11)	3.5 (0–55)	7 (0–48)	0.494
NOTCH 4	129 (68–279)	140 (71–330)	161 (48–369)	218 (107–419) &£	0.035
RBP-JK	12 (0–89)	4 (0–72)	10 (0–165) &	4 (0–124)	0.266
HES-1	89 (43–113)	69 (8–161)	79 (28–206)	131 (12–306)	0.176
HES-3	0 (0–7)	0 (0–6)	0 (0–8)	0 (0–5)	0.955
HES-5	281 (226–363)	267 (173–393)	293 (193–403)	317 (225–363) &	0.214
HES-6	0 (0–74)	0 (0–204)	0 (0–358)	2 (0–38)	0.662
HES-7	84 (21–184)	66 (0–229)	97 (0–181)	149 (97–226) *&£	0.003
HEY2	218 (153–252)	193 (97–306)	221 (116–339)	213 (2–336)	0.890
HEYL	24 (0–97)	43 (16–134)	51 (11–139)	50 (11–107)	0.773

Abbreviations: COPD, chronic obstructive pulmonary disease. Data are expressed as median (range). Statistics: The Kruskal–Wallis test was used for multiple comparisons, followed by the Mann–Whitney U-test for comparison between groups; *, *p* < 0.05, significantly different from control nonsmokers; &, *p* < 0.05, significantly different from control smokers with normal lung function; £, *p* < 0.05, significantly different from mild COPD. The exact “*p*” values for comparison between groups are given in the Results section (Section 2).

**Table 4:** Immunohistochemical quantification of Notch signaling proteins in the lung parenchyma.

Localization	Control Nonsmokers	Control Smokers	COPD Patients	Kruskal–Wallis *p*-Value
Bronchiolar Epithelium (score 0–3)				
JAGGED 1	0.0 (0.0)	0.0 (0.0)	0.0 (0.0)	0.928
JAGGED 2	0.5 (0.62)	0.81 (0.92)	0.5 (0.65)	0.528
DELTA 1	0.0 (0.19)	0.02 (0.25)	0.0 (0.0)	0.374
DELTA 4	0.17 (0.37)	0.37 (0.49)	0.15 (0.38)	0.425
NOTCH 1	0.0 (0.0)	0.26 (0.20) *	0.10 (0.25) *	**0.0075**
NOTCH 2	0.0 (0.15)	0.0 (0.20)	0.0 (0.0)	0.585
NOTCH 3	0.37 (0.36)	0.27 (0.35)	0.0 (0.41)	0.224
NOTCH 4	1.6 (1.0)	1.58 (0.75)	1.5 (0.67)	0.909
RBP-JK	0.0 (0.0)	0.0 (0.0)	0.0 (0.0)	0.654
HES-1	0.5 (1.31)	1.37 (1.12)	1.5 (1.40)	0.229
HES-3	1.0 (1.25)	0.68 (1.34)	0.54 (1.0)	0.626
HES-5	2.0 (0.55)	1.75 (1.07)	2.0 (0.35)	0.649
HES-6	0.0 (0.14)	0.0 (0.0)	0.0 (0.0)	0.658
HES-7	0.62 (0.77)	0.93 (1.32)	0.91 (0.59)	0.797
HEY2	0.60 (1.02)	0.25 (0.90)	0.50 (1.13)	0.992
HEYL	0.33 (0.40)	0.57 (0.63)	0.25 (0.35)	0.579
Bronchiolar Lamina Propria (score 0–3)				
JAGGED 1	0.0 (0.0)	0.0 (0.0)	0.0 (0.0)	nd
JAGGED 2	0.0 (0.12)	0.0 (0.10)	0.0 (0.0)	0.770
DELTA 1	0.0 (0.0)	0.0 (0.0)	0.0 (0.0)	nd
DELTA 4	0.0 (0.0)	0.0 (0.25)	0.0 (0.0)	0.210
NOTCH 1	0.0 (0.0)	0.0 (0.0)	0.0 (0.0)	0.882
NOTCH 2	0.0 (0.0)	0.0 (0.0)	0.0 (0.0)	nd
NOTCH 3	0.0 (0.0)	0.0 (0.0)	0. (0.0)	0.919
NOTCH 4	0.85 (0.50)	0.87 (0.48)	1.0 (0.2)	0.290
RBP-JK	0.0 (0.0)	0.0 (0.0)	0.0 (0.0)	nd
HES-1	0.05 (0.70)	0.75 (0.97)	0.5 (0.95)	0.324
HES-3	0.0 (0.0)	0.0 (0.0)	0.0 (0.0)	0.744
HES-5	1.0 (0.25)	0.5 (0.50)	1.0 (0.50)	0.708
HES-6	0.0 (0.0)	0.0 (0.0)	0.0 (0.0)	nd
HES-7	0.5 (0.25)	0.5 (0.72)	0.5 (0.21)	0.889
HEY2	0.0 (0.0)	0.0 (0.0)	0.0 (0.0)	nd
HEYL	0.0 (0.10)	0.20 (0.40)	0.0 (0.12)	0.385
Alveolar Macrophages (score 0–3)				
JAGGED 1	0.0 (0.0)	0.10 (0.24)	0.0 (0.11)	0.103
JAGGED 2	0.50 (0.88)	1.25 (1.41)	0.50 (0.55)	0.298
DELTA 1	0.0 (0.25)	0.87 (1.5)	0.05 (0.93)	0.148
DELTA 4	0.16 (0.5)	0.81 (0.70) *	0.62 (1.0)	**0.028**
NOTCH 1	0.37 (0.83)	0.72 (0.70)	0.50 (0.95)	0.553
NOTCH 2	0.0 (0.10)	0.37 (0.40) *	0.0 (0.11) &	**0.025**
NOTCH 3	0.10 (0.20)	0.33 (1.25)	0.17 (0.45)	0.107
NOTCH 4	1.25 (0.90)	1.5 (0.29)	1.5 (0.50)	0.817
RBP-JK	0.0 (0.0)	0.25 (0.50)	0.0 (0.50)	0.481
HES-1	0.75 (0.50)	1.0 (0.45)	0.85 (0.65)	0.283
HES-3	0.0 (0.09)	0.41 (0.88) *	0.0 (0.30)	0.051
HES-5	2.0 (0.62)	2.0 (1.12)	1.75 (0.50)	0.717
HES-6	0.0 (0.37)	0.25 (0.32)	0.0 (0.54)	0.606
HES-7	1.31 (1.05)	1.60 (1.20)	1.75 (0.79)	0.568
HEY2	0.55 (0.87)	0.74 (0.66)	0.50 (0.87)	0.621
HEYL	0.25 (0.40)	0.75 (0.56)	0.66 (0.62)	0.073
Alveolar Septa (score 0–3)				
JAGGED 1	0.0 (0.0)	0.0 (0.0)	0.0 (0.0)	nd
JAGGED 2	0.0 (0.50)	0.07 (0.50)	0.0 (0.22)	0.886
DELTA 1	0.0 (0.0)	0.0 (0.0)	0.0 (0.0)	nd
DELTA 4	0.0 (0.0)	0.0 (0.0)	0.0 (0.0)	0.991
NOTCH 1	0.0 (0.0)	0.0 (0.0)	0.0 (0.0)	0.810
NOTCH 2	0.0 (0.0)	0.0 (0.0)	0.0 (0.0)	0.903
NOTCH 3	0.0 (0.0)	0.0 (0.0)	0.0 (0.0)	0.927
NOTCH 4	1.0 (0.46)	1.2 (0.50)	1.37 (0.50)	0.550
RBP-JK	0.0 (0.0)	0.0 (0.0)	0.0 (0.0)	nd
HES-1	0.5 (0.62)	0.55 (0.40)	0.50 (0.58)	0.973
HES-3	0.0 (0.0)	0.0 (0.0)	0.0 (0.0)	nd
HES-5	1.0 (0.50)	1.0 (0.81)	0.75 (0.50)	0.532
HES-6	0.0 (0.0)	0.0 (0.0)	0.0 (0.0)	nd
HES-7	0.50 (0.33)	0.55 (0.62)	0.75 (0.33)	0.813
HEY2	0.0 (0.25)	0.0 (0.10)	0.0 (0.0)	0.663
HEYL	0.0 (0.0)	0.0 (0.0)	0.0 (0.0)	0.878
Lung vessels-(score 0–3)				
JAGGED 1	0.0 (0.0)	0.0 (0.0)	0.0 (0.0)	nd
JAGGED 2	0.5 (0.81)	0.75 (0.75)	0.25 (0.50)	0.182
DELTA 1	0.0 (0.0)	0.0 (0.0)	0.0 (0.0)	nd
DELTA 4	0.0 (0.0)	0.0 (0.0)	0.0 (0.0)	0.911
NOTCH 1	0.0 (0.0)	0.0 (0.0)	0.0 (0.0)	nd
NOTCH 2	0.0 (0.0)	0.0 (0.0)	0.0 (0.0)	0.903
NOTCH 3	0.0 (0.0)	0.0 (0.0)	0.0 (0.0)	0.918
NOTCH 4	1.5 (0.62)	1.5 (0.62)	1.5 (0.50)	0.712
RBP-JK	0.0 (0.0)	0.0 (0.0)	0.0 (0.0)	nd
HES-1	0.62 (0.75)	0.79 (0.87)	0.50 (1.17)	0.997
HES-3	0.0 (0.0)	0.0 (0.0)	0.0 (0.0)	0.907
HES-5	1.0 (0.50)	1.0 (0.62)	1.0 (0.50)	0.555
HES-6	0.0 (0.0)	0.0 (0.0)	0.0 (0.0)	nd
HES-7	0.50 (0.43)	0.35 (1.00)	0.75 (0.60)	0.936
HEY2	0.0 (0.50)	0.0 (0.5)	0.0 (0.0)	0.665
HEYL	0.0 (0.0)	0.0 (0.0)	0.0 (0.0)	nd

Abbreviations: COPD, chronic obstructive pulmonary disease. Data are expressed as median (IQR). Statistics: The Kruskal–Wallis test was used for multiple comparisons, followed by the Mann–Whitney U-test for comparison between groups; *, *p* < 0.05, significantly different from control nonsmokers; &, *p* < 0.05 and significantly different from control smokers with normal lung function. Lung vessels-e, endothelium in the lung vessels. The exact “*p*” values for comparison between groups are given in the Results section (Section 2).

## Data Availability

A preprint version of this manuscript is available on https://www.researchgate.net/publication/376142825_(accessed on 11 March 2024); Up-regulation_of_Notch_signaling_and_cell-differentiation_inhibitory_transcription_factors_in_the_lower_airways_of_stable_COPD_patients. The data from this study are available upon reasonable request.

## Supplementary Materials

The following supporting information can be downloaded at: https://www.mdpi.com/article/10.3390/ijms25063287/s1.

## Abbreviations

FEV1	Forced expiratory volume in one second
FVC	Forced vital capacity
MCOPD	Mild/moderate chronic obstructive pulmonary disease
SCOPD	Severe/very severe chronic obstructive pulmonary disease
CS	Control smokers with normal lung function
CNS	Control nonsmokers
Notch	Notch receptor
NICD	Notch-intracellular domain
DLL	Delta-like ligand
Jagged	Jag ligand
RBP-JK	Notch intracellular domain
HES	Hairy and enhancer of split
HEY2	Hairy/enhancer-of-split related with YRPW motif protein 2
HEYL	Hairy/enhancer-of-split related with YRPW motif-like protein

## References

[1] Bi P., Kuang S. (2015). Notch signaling as a novel regulator of metabolism. Trends Endocrinol. Metab..

[2] Collins B.J., Kleeberger W., Ball D.W. (2004). Notch in lung development and lung cancer. Semin. Cancer Biol..

[3] Hansson E.M., Lendahl U., Chapman G. (2004). Notch signaling in development and disease. Semin. Cancer Biol..

[4] Li X., Shu R., Filippatos G., Uhal B.D. (2004). Apoptosis in lung injury and remodeling. J. Appl. Physiol. (1985).

[5] Zong D., Ouyang R., Li J., Chen Y., Chen P. (2016). Notch signaling in lung diseases: Focus on Notch1 and Notch3. Ther. Adv. Respir. Dis..

[6] Hogg J.C., McDonough J.E., Suzuki M. (2013). Small airway obstruction in COPD: New insights based on micro-CT imaging and MRI imaging. Chest.

[7] Hogg J.C., McDonough J.E., Sanchez P.G., Cooper J.D., Coxson H.O., Elliott W.M., Naiman D., Pochettino M., Horng D., Gefter W.B. (2009). Micro-computed tomography measurements of peripheral lung pathology in chronic obstructive pulmonary disease. Proc. Am. Thorac. Soc..

[8] Vasilescu D.M., Martinez F.J., Marchetti N., Galbán C.J., Hatt C., Meldrum C.A., Dass C., Tanabe N., Reddy R.M., Lagstein A. (2019). Noninvasive Imaging Biomarker Identifies Small Airway Damage in Severe Chronic Obstructive Pulmonary Disease. Am. J. Respir. Crit. Care Med..

[9] Vasilescu D.M., Hackett T.L., Martinez F.J., Curtis J.L., Hogg J.C., Han M.K. (2020). Reply to Janssen and Wouters: Loss of Alveolar Attachments as a Pathomechanistic Link between Small Airway Disease and Emphysema. Am. J. Respir. Crit. Care Med..

[10] Tilley A.E., Harvey B.G., Heguy A., Hackett N.R., Wang R., O’Connor T.P., Crystal R.G. (2009). Down-regulation of the notch pathway in human airway epithelium in association with smoking and chronic obstructive pulmonary disease. Am. J. Respir. Crit. Care Med..

[11] Zong D., Li J., Cai S., He S., Liu Q., Jiang J., Chen S., Long Y., Chen Y., Chen P. (2018). Notch1 regulates endothelial apoptosis via the ERK pathway in chronic obstructive pulmonary disease. Am. J. Physiol. Cell Physiol..

[12] Boucherat O., Chakir J., Jeannotte L. (2012). The loss of Hoxa5 function promotes Notch-dependent goblet cell metaplasia in lung airways. Biol. Open.

[13] Guseh J.S., Bores S.A., Stanger B.Z., Zhou Q., Anderson W.J., Melton D.A., Rajagopal J. (2009). Notch signaling promotes airway mucous metaplasia and inhibits alveolar development. Development.

[14] Tsao P.N., Wei S.C., Wu M.F., Huang M.T., Lin H.Y., Lee M.C., Lin K.M., Wang I.J., Kaartinen V., Yang L.T. (2011). Notch signaling prevents mucous metaplasia in mouse conducting airways during postnatal development. Development.

[15] Hellström M., Phng L.K., Hofmann J.J., Wallgard E., Coultas L., Lindblom P., Alva J., Nilsson A.K., Karlsson L., Gaiano N. (2007). Dll4 signalling through Notch1 regulates formation of tip cells during angiogenesis. Nature.

[16] Siekmann A.F., Lawson N.D. (2007). Notch signalling limits angiogenic cell behaviour in developing zebrafish arteries. Nature.

[17] Hasan S.S., Tsaryk R., Lange M., Wisniewski L., Moore J.C., Lawson N.D., Wojciechowska K., Schnittler H., Siekmann A.F. (2017). Endothelial Notch signalling limits angiogenesis via control of artery formation. Nat. Cell Biol..

[18] Osborne B.A., Minter L.M. (2007). Notch signalling during peripheral T-cell activation and differentiation. Nat. Rev. Immunol..

[19] Ito T., Schaller M., Hogaboam C.M., Standiford T.J., Sandor M., Lukacs N.W., Chensue S.W., Kunkel S.L. (2009). TLR9 regulates the mycobacteria-elicited pulmonary granulomatous immune response in mice through DC-derived Notch ligand delta-like 4. J. Clin. Investig..

[20] Ito T., Allen R.M., Carson WF 4th Schaller M., Cavassani K.A., Hogaboam C.M., Lukacs N.W., Matsukawa A., Kunkel S.L. (2011). The critical role of Notch ligand Delta-like 1 in the pathogenesis of influenza A virus (H1N1) infection. PLoS Pathog..

[21] Ito T., Connett J.M., Kunkel S.L., Matsukawa A. (2012). Notch system in the linkage of innate and adaptive immunity. J. Leukoc. Biol..

[22] Di Stefano A., Rosani U., Levra S., Gnemmi I., Brun P., Maniscalco M., D’Anna S.E., Carriero V., Bertolini F., Ricciardolo F.L.M. (2023). Bone Morphogenic Proteins and Their Antagonists in the Lower Airways of Stable COPD Patients. Biology.

[23] Di Stefano A., Sangiorgi C., Gnemmi I., Casolari P., Brun P., Ricciardolo F.L.M., Contoli M., Papi A., Maniscalco P., Ruggeri P. (2018). TGF-β Signaling Pathways in Different Compartments of the Lower Airways of Patients With Stable COPD. Chest.

[24] Auderset F., Schuster S., Fasnacht N., Coutaz M., Charmoy M., Koch U., Favre S., Wilson A., Trottein F., Alexander J. (2013). Notch signaling regulates follicular helper T cell differentiation. J. Immunol..

[25] Radtke F., Fasnacht N., Macdonald H.R. (2010). Notch signaling in the immune system. Immunity.

[26] Vallese D., Ricciardolo F.L., Gnemmi I., Casolari P., Brun P., Sorbello V., Capelli A., Cappello F., Cavallesco G.N., Papi A. (2015). Phospho-p38 MAPK expression in COPD patients and asthmatics and in challenged bronchial epithelium. Respiration.

[27] Di Stefano A., Ricciardolo F.L.M., Caramori G., Adcock I.M., Chung K.F., Barnes P.J., Brun P., Leonardi A., Andò F., Vallese D. (2017). Bronchial inflammation and bacterial load in stable COPD is associated with TLR4 overexpression. Eur. Respir. J..

[28] Skokos D., Nussenzweig M.C. (2007). CD8- DCs induce IL-12-independent Th1 differentiation through Delta 4 Notch-like ligand in response to bacterial LPS. J. Exp. Med..

[29] Schaller M.A., Neupane R., Rudd B.D., Kunkel S.L., Kallal L.E., Lincoln P., Lowe J.B., Man Y., Lukacs N.W. (2007). Notch ligand Delta-like 4 regulates disease pathogenesis during respiratory viral infections by modulating Th2 cytokines. J. Exp. Med..

[30] Chen S., Zhou Y., Chen Y., Gu J. (2018). fastp: An ultra-fast all-in-one FASTQ preprocessor. Bioinformatics.

[31] Cozens A.L., Yezzi M.J., Kunzelmann K., Ohrui T., Chin L., Eng K., Finkbeiner W.E., Widdicombe J.H., Gruenert D.C. (1994). CFTR expression and chloride secretion in polarized immortal human bronchial epithelial cells. Am. J. Respir. Cell Mol. Biol..

