# Supplementary material for: Upregulation of Notch Signaling and Cell-Differentiation Inhibitory Transcription Factors in Stable Chronic Obstructive Pulmonary Disease Patients
Source: Int J Mol Sci. 2024 Mar 14;25(6):3287. doi: 10.3390/ijms25063287 (PMC10970547; doi:10.3390/ijms25063287)
Supplement: Supplementary file 1 [file ijms-25-03287-s001.zip › ijms-2874147-supplementary.pdf]

Supplementary Materials

Upregulation of Notch Signaling and Cell-Differentiation Inhibitory Transcription Factors in Stable Chronic Obstructive Pulmonary Disease Patients

Antonino Di Stefano [1,*], Isabella Gnemmi [1], Umberto Rosani [2], Mauro Maniscalco [3], Silvestro Ennio D'Anna [3], Paola Brun [4], Vitina Carriero [5], Francesca Bertolini [5], Bruno Balbi [1,6] and Fabio Luigi Massimo Ricciardolo [5]

[1] Divisione di Pneumologia and Laboratorio di Citoimmunopatologia dell'Apparato Cardio Respiratorio, Istituti Clinici Scientifici Maugeri, IRCCS, Respiratory Rehabilitation Unit of Gattico-Veruno, 28013 Novara, Italy
[2] Department of Biology, University of Padova, via U. Bassi 58/b, 35121 Padova, Italy
[3] Divisione di Pneumologia, Istituti Clinici Scientifici Maugeri, IRCCS, Telese, 82037 Benevento, Italy
[4] Department of Molecular Medicine, Histology Unit, University of Padova, 35121 Padova, Italy
[5] Department of Clinical and Biological Sciences, Severe Asthma and Rare Lung Disease Unit, San Luigi Gonzaga University Hospital, University of Turin, Orbassano, 10043 Turin, Italy
[6] Azienda Sanitaria Locale (ASL), 28100 Novara, Italy

Methods

Subjects

All the COPD and healthy control subjects were recruited from the Respiratory Medicine Unit of the "Istituti Clinici Scientifici Maugeri" (Veruno, Italy) and the San Luigi Gonzaga University Hospital (Orbassano-Turin, Italy). Archival material was used in the present study (**S1**). We obtained bronchial biopsies from 58 subjects for the immunohistochemical study of bronchial biopsies. The characteristics of these subjects are reported in **Table 1**. Thirty-three subjects undergoing lung resection for a solitary peripheral neoplasm were recruited for the immunohistochemical study of peripheral lung tissue and transcriptomic analysis of bronchial rings and lung tissue. The characteristics of these subjects are reported in **Table 2**.

All the COPD patients were stable and none of the COPD subjects were treated with theophylline, antibiotics, antioxidants, mucolytics, and/or glucocorticoids in the month prior to bronchoscopy or lung resection surgery. The study conformed to the Declaration of Helsinki and the study was approved by the Institutional Review Boards of Istituti Clinici Scientifici Maugeri (protocol p112).

Lung function tests and volumes

The pulmonary function tests included measurements of FEV_1 and FEV_1/FVC under baseline conditions in all the subjects examined (6200 Autobox Pulmonary Function Laboratory; Sensormedics Corp., Yorba Linda, CA). In order to assess the reversibility of airflow obstruction and post bronchodilator functional values the FEV_1 and $FEV_1/FVC\%$ measurements in the groups of subjects with $FEV_1/FVC\% \leq 70\%$ pre-bronchodilator was repeated 20 min after the inhalation of 0.4 mg of salbutamol. Pulmonary function tests were performed on all subjects, according to current guidelines. The severity of the airflow obstruction in COPD patients was staged using GOLD criteria [https://goldcopd.org/wp-content/uploads/2021/12/GOLD-REPORT-2022-v1.1-22Nov2021_WMV.pdf] on the basis of pulmonary function tests.

Fiberoptic Bronchoscopy, Collection and Processing of Bronchial Biopsies

Subjects attended the bronchoscopy suite at 8.30 am. after having fasted from midnight and were pre-treated with atropine (0.6 mg IV) and midazolam (5-10 mg IV). Oxygen (3 l/min) was administered via nasal prongs throughout the procedure and oxygen saturation was monitored with a digital oximeter. Using local anesthesia with lidocaine (4%) to the upper airways and larynx, a fiberoptic bronchoscope (Olympus BF10 Key-Med, Southend, UK) was passed through the nasal passages into the trachea. Further lidocaine (2%) was sprayed into the lower airways, and four bronchial biopsy specimens were taken from segmental and subsegmental airways (4th to 6th airway generation) of the right lower and upper lobes using size 19 cupped forceps. Bronchial biopsies for immunohistochemistry were gently extracted from the forceps and processed for light microscopy (**22**). At least two samples were embedded in Tissue Tek II OCT (Miles Scientific,

Naperville, IL), frozen within 15 min in isopentane pre-cooled in liquid nitrogen, and stored at –80°C. The best frozen sample was then orientated and 6μm thick cryostat sections were cut for immunohistochemical light microscopy analysis and processed as described below.

Collection and Processing of the Peripheral Lung Tissue

Thirty-three subjects undergoing lung resection surgery for a solitary peripheral neoplasm were recruited. Ten were non-smokers with normal lung function, 10 subjects were smokers with normal lung function and 13 were smokers with COPD (**Table 2**). All former smokers had stopped smoking for more than one year. None of the subjects had undergone preoperative chemotherapy and/or radiotherapy, nor had they been treated with bronchodilators, theophylline, antibiotics, antioxidants and/or glucocorticoids in the month prior to surgery. Lung tissue processing was performed as previously described (**22, 23**). Two to four randomly selected tissue blocks and one-two bronchial rings taken from the lungs, were obtained during surgery, avoiding areas grossly invaded by tumour. The samples were fixed in 4% formaldehyde in phosphate-buffered saline at pH 7.2 or frozen in liquid nitrogen. Fixed specimens, after dehydration, were embedded in paraffin wax. Serial sections 6 μm thick from frozen specimens were first cut and stained with haematoxylin-eosin (H&E) in order to visualize the morphology and to exclude the presence of microscopically evident tumour infiltration. Frozen tissue specimens were used in this study. Specimens were then cut for immunohistochemical analysis and were placed on charged slides as previously reported (**22,23**).

Immunohistochemistry of OCT-Embedded Bronchial Biopsies

Sections from each sample were stained with antibodies specific for Notch signaling molecules, their ligands and their effector transcription factors (**Table S1**). Briefly, after blocking non-specific binding sites with serum derived from the same animal species as the secondary antibody, the primary antibody was applied at optimal dilutions in TRIS-buffered saline (0.15 M saline containing 0.05 M TRIS-hydrochloric acid at pH 7.6) and incubated 1hr at room temperature in a humid chamber. Antibody binding was detected with secondary anti-mouse (Vector, BA 2000), anti-rabbit (Vector, BA 1000) or anti-goat (Vector, BA 5000) antibodies followed by ABC kit AP AK5000, Vectastain and fast-red substrate (red color) or ABC kit HRP Elite, PK6100, Vectastain and diaminobenzidine substrate (brown color). Nasal polyp sections or human tonsils were used as positive controls. For the negative control, normal goat (sc—2048), mouse (sc-2025) or rabbit (sc-2027) non-specific immunoglobulins (Santa Cruz Biotechnology, Santa Cruz, CA, USA) were used at the same protein concentration as the primary antibody.

Immunohistochemistry in Human Peripheral Lung Tissue

Immunostaining of frozen peripheral lung tissue was performed as previously described (**22,23**). In the present study frozen sections were used for immunohistochemical analysis.

Endogenous peroxidase activity was blocked by incubating slides in 3% hydrogen peroxide (H_2O_2) in phosphate-buffered saline (PBS) followed by washing in PBS. Cell membranes were permeabilised adding 0.1% saponin to the PBS. Non-specific labeling was blocked by coating with blocking serum (5% normal animal serum) for 20 minutes at room temperature. After washing in PBS, the sections were incubated with primary antibodies used for the bronchial biopsies (**Table S1**). Control slides were included in each staining run using human tonsils or nasal polyps as a positive control for all the immunostaining performed. Slides were then incubated with chromogen-fast diaminobenzidine (DAB) as chromogenic substance. After which they were counterstained in haematoxylin and mounted on permanent mounting media.

Scoring System for Immunohistochemistry in the Bronchial Biopsies

Light-microscopic analysis was performed at a magnification of 630x.

The immunostaining for all the antigens studied was scored (range: 0 = absence of immunostaining to 3 = extensive intense immunostaining) in the intact bronchial epithelium. The final result was expressed as the average of all scored fields performed in each biopsy.

The immunostained cells in the bronchial lamina propria were quantified 100µm beneath the epithelial basement membrane in several non-overlapping high-power fields until the whole specimen was examined. The final result was expressed as the number of positive cells per square millimeter.

Scoring System for Immunohistochemistry in the Peripheral Lung Tissue

All disposable bronchioles, alveolar macrophages, alveolar septa and vessels observed in each lung section specimen were analyzed for each immunostained section. The immunopositivity was scored (0, absence of immunostaining, 1, 33% of immunostained cells, 2, 66% of immunostained cells, 3, almost all positive cells. Intensity of immunopositivity was considered adding a 0.5 score point to the established score applied on the basis of number of positive cells in the bronchiolar epithelium, bronchiolar lamina propria, alveolar macrophages, alveolar septa and lung vessels (**22,23**).

ELISA tests in the peripheral lung tissue homogenates

DLL1 (MyBioSource, Cat. N. MBS918959, lower detection limit, 7.8 pg/mL), DLL4 (MyBioSource, Cat. N. MBS452599, lower detection limit, 0.054 ng/mL), Notch1 (Cloud Clone Corp., Cat. N. SEG797Hu, lower detection limit, 0.060 ng/mL), Notch2 (MyBioSource, Cat. N. MBS455626, lower detection limit, 0.33 ng/mL), Notch4 (MyBioSource, Cat. N. MBS166620, lower detection limit, 14.7 ng/L), HES1 (MyBioSource, Cat. N. MBS7203329, lower detection limit, 1.0 pg/mL), HES5 (Abbexa, Cat. N. abx387776, lower detection limit, 0.05 ng/mL), HES7 (Abbexa, Cat. N. abx527037, lower detection limit, 37 pg/mL), protein quantification was performed in the lung tissue homogenates obtained from frozen tissue specimens which were also used for immunohistochemical analysis. All ELISA kits were used according to the manufacturer's instructions.

RNA extraction and sequencing from bronchial rings and lung specimens

Frozen Lung parenchymal tissue used for immunohistochemical analysis and bronchial rings taken from the same patients were also used for RNA extraction, sequencing and gene expression data analysis.

RNA extraction was performed with the RNAeasy micro kit (Qiagen, Hilden, Germany) following manufacturer instructions. A DNA removal step was applied using 500 units of RNase-free DNase (Qiagen) at room temperature for 15 minutes. Total RNA was resuspended in RNase-free water (Thermo Fisher, Carlsbad, US) and the RNA/DNA concentrations in each sample were quantified using the Qubit RNA and DNA high-sensitivity Assay Kit (Thermo Fisher). RNA qualities were assessed with an Agilent Bioanalyzer 2100 equipped with a RNA nano 6000 kit (Agilent, Santa Clara, CA, USA). Due to the low RIN values obtained for lung parenchyma samples, RNA-sequencing libraries for these samples were prepared following a 3'-end sequencing procedure using the QuantSeq 3' mRNA-Seq Library Prep Kit FWD for Illumina (Lexogen, Vienna, Austria). Consequently, lung parenchyma libraries were sequenced using an Illumina NextSeq500 (Cribi, UniPD, Padova, Italy) with a 75 single end read layout. Since, no quality issues were encountered for the bronchial ring samples, the standard Illumina library preparation were performed. Bronchial ring libraries were then sequenced with a 150 paired-end read layout (Cribi, UniPD, Padova, Italy).

Data analysis of RNA-seq data

The raw Illumina reads were trimmed for quality using *fastp* (**30**), setting a minimal Phred quality of 25 and removing the sequencing adaptors. Raw Illumina datasets have been submitted to the NCBI Short Read Archive (SRA) under the project ID PRJNA1041288. FASTQ files were imported in the CLC Genomic Workbench v.21 (Qiagen, Hilden, Denmark) and analyzed as follows: To identify differentially expressed genes (DEGs) the trimmed reads were mapped on the human reference genome (hg19, Ensembl v.99) applying the following parameters: Mismatch cost=2; Insertion cost=3; Deletion cost=3; Length fraction=0.8; Similarity fraction=0.8 and strand-specific mapping. Expression values were counted as Transcripts Per Millions (TPMs). A Baggerley test with false discovery rate (FDR) p-value correction was applied to identify differentially expressed genes (DEGs), setting a cutoff of 2-Fold changes (FC) and a 0.05 of FDR p-value. Limited to the gene of interest, the expression levels were extracted from the overall dataset and further discussed.

Cell culture and treatments

We used the SV40 large T antigen-transformed 16HBE cell line which retains the differentiated morphology and function of normal human bronchial epithelial cells (NHBE) (**31**) 16HBE cells were maintained in Dulbecco's modified minimum essential medium (DMEM), supplemented with 10% v/v fetal bovine serum (FBS), 50 IU/ml penicillin, 50 µg/ml streptomycin, 1x non-essential amino acids, 1mM sodium pyruvate and 2mM glutamine (37°C, 5% CO2) (**31**). When cells were 60-70% confluent, the complete medium was replaced with DMEM with 1% FBS for starvation time (24 h). 16HBE cells were cultured for 0-24 h because of their lower resistance to starvation. Non-treated 16HBE cells were used as controls. All experiments were performed in quadruplicate, i.e. four independent experiments for each type of treatment (LPS, Sigma L9143, 10 µg/ml; H_2O_2, Sigma-Aldrich 18304, 100µM)) and each time exposure (4-12-24h).

ELISA tests in the cell lysates of LPS and H_2O_2 treated and unreated 16HBE cells

DLL1, DLL4, Notch1, Notch2, Notch4, HES1, HES5 and HES7 proteins were also analyzed in treated and untreated 16HBE cells using the same ELISA kits as reported for peripheral lung tissue homogenates. Data obtained are reported in results section. Kits were used according to the manufacturer's instructions.

Statistical analysis applied to functional and morphological data

Group data were expressed as mean (standard deviation) for functional data or median (range) or interquartile range (IQR) for morphologic data. Differences between groups were analyzed using analysis of variance (ANOVA) for functional data. ANOVA was followed by an unpaired t-test for comparison between groups. The KruskalWallis test was applied to the morphologic data followed by a Mann-Whitney U-test for comparison between groups. In vitro data were analyzed by the Mann-Whitney U test. Correlation coefficients were calculated using the Spearman rank method. Probability values of $p<0.05$ were considered significant. Data analysis was performed using the Stat View SE Graphics program (Abacus Concepts Inc., Berkeley, CA-USA.

Table S1. Primary antibodies and immunohistochemical conditions used for identification of Notch signaling proteins in bronchial biopsies and peripheral lung.

Target	Supplier	Cat.#[a]	Source	Dilution	Positive control
JAGGED 1	R&D	AF1277	Goat	1:100	Nasal polyp, human tonsil
JAGGED 2	R&D	AF1726	Goat	1:200	Nasal polyp, human tonsil
DELTA 1	R&D	MAB18181	Mouse	1:50	Nasal polyp, human tonsil
DELTA 4	S. Cruz	SC18640	Goat	1:100	Nasal polyp, human tonsil
NOTCH 1	R&D	MAB3647	Mouse	1:150	Nasal polyp, human tonsil
NOTCH 2	R&D	AF3735	Goat	1:100	Nasal polyp, human tonsil
NOTCH 3	S. Cruz	SC7424	Goat	1:100	Nasal polyp, human tonsil
NOTCH 4	R&D	AF3847	Goat	1:25	Nasal polyp, human tonsil
RBP-JK	R&D	AF4079	Goat	1:100	Nasal polyp, human tonsil
HES-1	R&D	AF3317	Goat	1:40	Nasal polyp, human tonsil
HES-3	ATLAS	HPA047927	Rabbit	1:100	Nasal polyp,

					human tonsil
HES-5	Novus Biol.	N-BP2-27174	Rabbit	1:25	Nasal polyp, human tonsil
HES-6	Santa Cruz	SC133196	Mouse	1:100	Nasal polyp, human tonsil
HES-7	Santa Cruz	SC133652	Rabbit	1:50	Nasal polyp, human tonsil
HEY2	ATLAS	HPA030205	Rabbit	1:50	Nasal polyp, human tonsil
HEYL	ATLAS	HPA001438	Rabbit	1:100	Nasal polyp, human tonsil

[a]Cat#, catalogue number

S-Results

Table S2: Gene expression levels of 16 selected genes related to the NOTCH pathway. For each gene, the expression levels in rings and lung parenchyma of control non-smokers, control smokers and COPD patients were reported together with fold-change and p-value for the different comparisons. Statistically significant comparisons are shown in bold type..

RINGS

	CS vs COPD		CNS vs COPD		CNS vs CS		Mean expression (TPMs)		
Gene ID	FC	P-value	FC	P-value	FC	P-value	CNS	COPD	CS
DLL1	1.94	0.06	-2.15	0.03	**-4.18**	**3.59E-05**	3.23	19.36	25.87
DLL4	1.06	0.91	-2.22	0.1	-2.34	0.08	0.91	4.24	4.4
HES1	1.39	0.26	-1.35	0.32	-1.88	0.03	23.77	55.51	87.28
HES3							0	0	0
HES5	-4.49	0.12	**-46.08**	**0.01**	-10.27	0.13	0	0.37	0.13
HES6	-1.33	0.38	1.33	0.38	1.78	0.08	11.34	11.94	13.53
HES7	1.84	0.49	-2.46	0.39	-4.51	0.14	0.02	0.08	0.15
HEY2	-1.18	0.61	-1.11	0.75	1.06	0.85	3.19	5.79	6.23
HEYL	-1.07	0.89	-1.65	0.26	-1.55	0.33	3.43	12.25	11.46
JAG1_2							0	0	0
JAG2	1.53	0.1	-1.49	0.12	**-2.28**	**1.42E-03**	3.82	11.15	18.27
NOTCH1	1.7	0.12	**-2.67**	**3.80E-03**	**-4.53**	**8.48E-06**	1.25	7.47	12.79
NOTCH2	1.19	0.47	-1.48	0.11	-1.77	0.02	3.42	9.25	13.45
NOTCH3	1.04	0.91	-1.89	0.03	-1.96	0.03	5.78	23.3	25.95
NOTCH4	1.21	0.55	**-2.51**	**5.34E-03**	**-3.05**	**7.31E-04**	0.89	4.95	6.16
RBPJL							0	0	0

LUNG parenchyma

	CS vs COPD		CNS vs COPD		CNS vs CS		Mean expression (TPMs)		
Gene ID	FC	P-value	FC	P-value	FC	P-value	CNS	COPD	CS
DLL1	-1.4	0.27	1.09	0.78	-1.53	0.13	7.62	4.85	5.64
DLL4	1.04	0.92	1.41	0.32	-1.36	0.35	4.99	3.58	5.37
HES1	1.2	0.52	1.13	0.67	1.07	0.81	167.84	184.55	214.79
HES3							0	0	0
HES5	-1.88	0.4	1.05	0.95	-1.98	0.32	0.53	0.23	0.26
HES6	-1.13	0.69	1.1	0.76	-1.24	0.45	5.04	3.89	4.45

HES7	-2.36	0.29	-3.3	0.13	1.4	0.59	0.21	0.3	0.1
HEY2	-1.32	0.41	1.12	0.73	-1.48	0.21	6.31	4.32	4.84
HEYL	1	0.99	1.01	0.98	-1	0.99	24.57	24.21	26.07
JAG1_2							0	0	0
JAG2	-1.08	0.69	-1.15	0.49	1.06	0.76	17.03	18.67	16.63
NOTCH1	1.07	0.7	1.24	0.17	-1.17	0.3	38.93	32.65	42.65
NOTCH2	-1.22	0.19	-1.02	0.9	-1.2	0.19	62.39	50.98	52.4
NOTCH3	1.1	0.62	1.05	0.8	1.05	0.78	46.88	49.95	54.55
NOTCH4	1.34	0.29	1.31	0.31	1.02	0.94	43.09	44.51	61.12
RBPJL							0	0	0

CNS, control non-smokers; CS, control smokers; COPD, patients with chronic obstructive pulmonary disease

References

22. Di Stefano A, Rosani U, Levra S, Gnemmi I, Brun P, Maniscalco M, D'Anna SE, Carriero V, Bertolini F, Ricciardolo FLM. Bone Morphogenic Proteins and Their Antagonists in the Lower Airways of Stable COPD Patients. Biology. 2023; 12(10): 1304. doi: 10.3390/biology12101304

23. Di Stefano A, Sangiorgi C, Gnemmi I, Casolari P, Brun P, Ricciardolo FLM, Contoli M, Papi A, Maniscalco P, Ruggeri P, Girbino G, Cappello F, Pavlides S, Guo Y, Chung KF, Barnes PJ, Adcock IM, Balbi B, Caramori G. TGF-β Signaling Pathways in Different Compartments of the Lower Airways of Patients With Stable COPD. Chest. 2018 Apr;153(4):851-862. doi: 10.1016/j.chest.2017.12.017.

30. Chen S, Zhou Y, Chen Y, Gu J. fastp: an ultra-fast all-in-one FASTQ preprocessor. Bioinformatics. 2018 Sep 1;34(17):i884-i890. doi: 10.1093/bioinformatics/bty560. PMID: 30423086 Bioinformatics, 34, 2018, i884–i890 doi: 10.1093/bioinformatics/bty560

31. Cozens AL, Yezzi MJ, Kunzelmann K, Ohrui T, Chin L, Eng K, et al. CFTR expression and chloride secretion in polarized immortal human bronchial epithelial cells. Am J Respir Cell Mol Biol. 1994;10:38-47.
